# Incidence and Pathogenicity of *Yersinia pseudotuberculosis* and *Yersinia enterocolitica* in a Large Rhesus Macaque (*Macaca mulatta*) Colony (2000–2024)

**DOI:** 10.3390/microorganisms14030596

**Published:** 2026-03-06

**Authors:** Sheena Haney, Anne D. Lewis, Hilary Ann Lakin

**Affiliations:** 1 Division of Comparative Medicine, Oregon National Primate Research Center, Oregon Health & Science University, Beaverton, OR 97006, USA; haneys@ohsu.edu; 2 Carlson College of Veterinary Medicine, Oregon State University, Corvallis, OR 97331, USA; hlakin@purdue.edu

**Keywords:** *Yersinia pseudotuberculosis*, *Yersinia enterocolitica*, nonhuman primates, *Macaca mulatta*, epidemiology, pathogenicity, zoonoses, colony health surveillance, necropsy, histopathology

## Abstract

Comprehensive epidemiological reports on the incidence and pathogenicity of *Yersinia* spp., specifically *Yersinia pseudotuberculosis* and *Yersinia enterocolitica*, in rhesus macaques (*Macaca mulatta*) are not prevalent. Here we report findings from a retrospective analysis of microbial culture results, necropsy reports, and histology records collected over 24 years (2000–2024) in a large captive rhesus macaque colony at the Oregon National Primate Research Center (ONPRC). Findings are compared between animals infected with either *Y. pseudotuberculosis* or *Y. enterocolitica* to evaluate the prevalence of infection within the population and determine whether *Y. pseudotuberculosis* is more likely to be associated with systemic disease and higher mortality than *Y. enterocolitica*. Among 101 affected animals, *Y. pseudotuberculosis* was the predominant species (75.2%), followed by *Y. enterocolitica* (22.7%) and *Y. kristensenii* (2.0%). Overall mortality among animals with confirmed yersiniosis was 25.3%, with comparable mortality rates for *Y. pseudotuberculosis* (23.7%) and *Y. enterocolitica* (30.4%) infections. *Y. pseudotuberculosis* was most frequently associated with systemic illness, spontaneous death, or significant clinical decline. Overall, these findings emphasize the importance of continued surveillance and targeted management strategies to mitigate the impact of *Yersinia* infections in captive nonhuman primate colonies.

## 1. Introduction

*Yersinia enterocolitica* and *Yersinia pseudotuberculosis* are Gram-negative, facultatively anaerobic coccobacilli in the family Enterobacteriaceae. Of the 26 species of *Yersinia*, *enterocolitica* and *pseudotuberculosis* are commonly recognized zoonotic pathogens capable of causing gastrointestinal and systemic disease in a variety of species, including nonhuman primates (NHPs) and humans [1,2]. In both human and NHP populations, these enteropathogenic *Yersiniae* are commonly identified causative agents of yersiniosis [3,4]. Old and New World monkey species, including squirrel monkeys (*Samiri* spp.), marmosets (*Callithrix* spp.), brown-headed spider monkeys (*Ateles fusciceps*), owl monkeys (*Aotus* spp.), patas monkeys (*Erythrocebus patas*), vervets (*Chlorocebus* spp.), mangabeys (*Cercocebus* spp.), tamarins (*Saguinus* spp.), and macaques (*Macaca fascicularis* and *Macaca mulatta*), are reported to be sensitive to *Yersinia* infection and express variable morbidity and mortality [5,6,7]. 

Notable similarities and differences exist between humans and NHPs with yersiniosis. In humans, *Y. pseudotuberculosis* infections are less common than *Y. enterocolitica* but tend to cause more mesenteric lymphadenitis and appendicitis-like presentations, sometimes progressing to systemic disease, notably in vulnerable populations such as children or those with underlying conditions. Approximately 75% of patients infected with *Y. pseudotuberculosis* are between the ages of five and 15 years [8]. *Y. pseudotuberculosis* is considered the evolutionary ancestor of *Y. pestis*, highlighting its historic relevance as a zoonotic agent [9]. In contrast, *Y. enterocolitica* is more frequently associated with gastroenteritis, including diarrhea, abdominal pain, and fever, and it more often leads to pseudoappendicitis. Transmission typically occurs after consumption of contaminated food, with pork being a common source [10,11]. Certain *Y. enterocolitica* serotypes (O:3, O:9, O:8, and O:5,27) are most commonly associated with severe gastrointestinal disease [12]. Sepsis in infants and immunocompromised patients is associated with high mortality rates [11]. While both pathogens can cause extraintestinal complications like reactive arthritis or erythema nodosum in humans, *Y. pseudotuberculosis* is more commonly linked to systemic involvement, while *Y. enterocolitica* is notable for its higher incidence, prolonged fecal shedding, and the potential for severe outcomes, such as sepsis, in immunocompromised individuals [8,11]. 

In captive NHPs, including rhesus macaques (*Macaca mulatta*), both species of enteropathogenic *Yersinia* have been described as causing comparable clinical symptoms [13,14]. Infections with *Y. pseudotuberculosis* and *Y. enterocolitica* are often associated with enterocolitis and manifest clinically as self-limiting diarrhea (with or without blood), dehydration, anorexia, depression, and weight loss. Less frequently, these pathogens can cause systemic infection and septicemia, which carry significant morbidity and mortality [15,16]. In these cases, gross lesions may include ulcerative, necrotizing enterocolitis, with discrete multifocal mucosal ulcers within the small intestine and colon, and enlarged, edematous mesenteric lymph nodes. Bacterial dissemination to extraintestinal organs, especially the liver and spleen, may occur [6]. The likelihood of disease expression may be influenced by predisposing factors such as physiological stress, immunosuppression, and concurrent infections. Coinfections with other enteropathogens (e.g., *Campylobacter* spp., *Shigella* spp., intestinal parasites) may exacerbate clinical disease [7,17]. While both organisms produce similar clinical conditions, *Y. enterocolitica* tends to remain localized to the gastrointestinal tract, while *Y. pseudotuberculosis* is more frequently associated with extraintestinal dissemination [7,18]. Reproductive effects, including abortion and stillbirths, secondary to *Y. pseudotuberculosis* infection have also been described [13]. 

Diagnosis of yersiniosis typically relies on a combination of clinical signs, microbiological culture, and histopathologic evaluation. Culture obtained from feces, intestinal tissue, blood, or mesenteric lymph nodes remains the gold standard. *Yersinia* spp. grow on blood, chocolate, MacConkey, and other laboratory media between 4 °C and 43 °C. The slow-growing nature of *Yersinia* spp. can present challenges, especially when there is competition with commensal flora [6,15]. Leukocytosis secondary to neutrophilia, hyponatremia, hypochloremia, prerenal azotemia, and moderate hyperfibrinogenemia have been reported [19]. 

Transmission typically occurs via the fecal-oral route. Natural reservoirs include wild rodents, birds, and contaminated water sources [14]. Asymptomatic carriage and intermittent shedding in NHP colonies are suspected but poorly characterized, complicating eradication and surveillance strategies [13,20]. Subclinical carriers may serve as reservoirs of infection, intermittently introducing *Yersinia* spp. into social groups without overt clinical signs. This has been documented in other enteric pathogens of NHPs, where stress, dietary changes, or concurrent infections can trigger bacterial shedding and facilitate transmission [13,21,22]. Environmental persistence also plays a role in outbreaks, as both *Y. pseudotuberculosis* and *Y. enterocolitica* are psychrotrophic organisms with a known resistance to cold temperatures and the ability to survive for prolonged periods in soil and water [8,23]. Clinical disease is most commonly observed in outdoor-housed NHPs during cooler months, particularly winter and early spring [18]. 

The significance of these pathogens has been well documented in veterinary and public health contexts [24,25,26,27], and it is widely acknowledged that, in NHP facilities, outbreaks can compromise research programs, animal welfare, and breeding efforts, while posing zoonotic risks to animal care staff and researchers [4,16,26,27]. Despite this importance, comprehensive epidemiological data characterizing the incidence, clinical presentation, and pathogenic outcomes of yersiniosis in captive rhesus macaques remain limited, leaving critical gaps in understanding of disease dynamics and control in NHP populations. 

In this study, we retrospectively analyze 24 years of microbiological, clinical, and histopathology data from the Oregon National Primate Research Center (ONPRC) to better understand the epidemiology and pathogenic variation in *Yersinia* infections in a large colony of rhesus macaques. Specifically, we aim to compare the frequency and clinical severity of infections caused by *Y. pseudotuberculosis* versus *Y. enterocolitica*, with particular attention to differences in clinical signs, necropsy findings, and histopathologic outcomes. This work seeks to enhance our understanding of the prevalence and severity of these important pathogens in captive primate populations.

## 2. Materials and Methods

All animals from this study were housed at the ONPRC, an Association for Assessment and Accreditation of Laboratory Animal Care International (AAALACi) accredited facility. This was a retrospective study utilizing clinical and diagnostic records generated at the ONPRC. No animals were experimentally infected for the purposes of this study. All colony management practices were overseen by the ONPRC Institutional Animal Care and Use Committee (IACUC).

Microbial culture results, animal demographic and clinical information, and necropsy records were obtained from the ONPRC electronic health record system, Primate Records and Information Management (PRIMe). The PRIMe database is a centralized, searchable electronic system that contains clinical exam notes, laboratory test results, diagnostic imaging, housing assignments, and necropsy reports. Data is entered by veterinary staff and pathologists and undergoes periodic quality review. Health and colony management records for 21,510 rhesus macaques within the designated study timeframe (January 2000 through December 2024) were available for review. The colony included animals of both sexes and a broad age distribution, maintained in a variety of housing environments including indoor, outdoor, and indoor/outdoor facilities. Age categories were defined as neonatal, (0–28 days), infant (29–364 days), juvenile (1 year to 3 years), adult (4 years to 17 years), and geriatric (18 years or older). Animals were socially housed whenever possible, and husbandry practices followed standard ONPRC protocols for primate care, feeding, and sanitation. 

To identify cases of *Yersinia* spp. infection, microbiology records in PRIMe were queried for all cultures collected between 2000 and 2024 that were positive for *Yersinia*. Available results included cultures positive for *Y. enterocolitica*, *Y. pseudotuberculosis*, and *Y. kristensenii*. Culture results from animals treated clinically were obtained from fresh fecal swabs. Cultures collected at time of necropsy included gastrointestinal contents, and sectioned surfaces of lymph nodes, and other viscera. Each culture had been submitted to the ONPRC Clinical Pathology Laboratory as part of routine veterinary diagnostics performed on live, clinically healthy and ill animals, or necropsy evaluations. Cultures were incubated on MacConkey agar at room temperature for 24–48 h to limit overgrowth by non-target bacteria. Subcultures of all non-lactose fermenting colonies, identified within 48 h, were incubated on blood agar plates at 37 °F for 24 h. Finally, a urease tube was inoculated with the pure culture using aseptic technique. Identification of *Yersinia* species was performed on colonies appearing within 4–24 h, using the API^®^ 20E bacterial identification system, developed by BioMerieux (Marcy-l’Étoile, France). Due to the retrospective nature of this study, the use of molecular techniques to further characterize the isolated *Yersinia* strains was not available. Duplicate culture results from the same specimen were excluded, and when multiple positive cultures were identified for a single animal, all were recorded and linked under the unique animal identifier. 

For each animal with a positive culture, associated clinical records were reviewed to extract demographic data (sex, age, housing type, season at time of microbial culture), presenting clinical signs, diagnostic procedures, and treatment information when available. Necropsy reports were also reviewed for gross and histologic findings consistent with *Yersinia* infection. All pathology reports were authored or reviewed by diplomates of the American College of Veterinary Pathologists. Full necropsies and standard tissue collection of major organs for each animal had been performed. Tissues were preserved in 10% neutral buffered formalin and routinely processed into paraffin, sectioned at 4–6 µm, and stained with hematoxylin and eosin (H&E).

Differences in mortality, rate of spontaneous death (animals found deceased without prior clinical signs), and incidence of co-infection between rhesus macaques infected with *Y. pseudotuberculosis* and *Y. enterocolitica* were assessed using two-sided Fisher’s exact test. Relative risk (RR) and odds ratio (OR) with 95% confidence intervals (CI) were calculated. *p* < 0.05 was considered statistically significant. All analyses were performed using the GraphPad QuickCalcs online contingency table calculator (GraphPad Software, La Jolla, CA, USA; https://www.graphpad.com, accessed on 9 December 2025). 

## 3. Results

Between the years 2000 and 2024, there were 113 positive samples for *Yersinia* spp. from 101 individual rhesus macaques at the ONPRC. Samples were either collected upon presentation to the colony clinic or at necropsy. Of these 101 animals, 76 (75.2%) animals cultured positive for *Y. pseudotuberculosis* and 23 (22.7%) for *Y. enterocolitica*. Two animals cultured positive for *Y. kristensenii* (1.98%). 

All positive culture results originated from animals housed in outdoor or indoor/outdoor facilities. There were seven non-consecutive years that no *Yersinia* spp. were cultured in rhesus macaques at the ONPRC. This included 2003–2006, 2010, 2014, and 2018. Totals for all other years ranged from one case to 24 cases per year. Most cases occurred in the winter months, defined as December through February (n = 58, 57.4%), followed by the spring months, defined as March through May (n = 33, 32.7%). Fewer cases were detected in the summer months, defined as June through August (n = 1, 0.99%), and the fall months, defined as September through November (n = 9, 8.9%). Mortality rates were highest in the winter (n = 14, 13.9%) with *Y. pseudotuberculosis* cases presenting to necropsy 10 times and *Y. enterocolitica* cases presenting to necropsy 4 times throughout the winter months. Clinical cases (those animals treated for infection and successfully released from care) presenting with *Y. pseudotuberculosis* occurred most commonly during the winter (n = 35, 34.7%), while clinical cases presenting with *Y. enterocolitica* occurred more frequently during the spring months (n = 9, 8.9%). A summary of all culture results organized by year can be found in Table A2 of Appendix B.

Clinical Presentation:

During this 24-year period, *Yersinia* spp. were isolated from 76 animals that were treated in the ONPRC colony clinic and successfully released from care. Animals that did not survive are addressed in the following section. *Yersinia pseudotuberculosis* was isolated from 58 (76.3%) of these animals, while *Y. enterocolitica* was isolated from 16 (21.1%). *Y. kristensenii* was isolated from two animals (2.6%). Due to its low prevalence and absence of clinical significance, limited attention was given to *Y. kristensenii* during this study, and it was excluded from all statistical analyses. Of these rhesus macaques, 43 (56.6%) were females, and 33 were males (43.4%), ranging in age from 13 days to 16 years. The age categories of those affected included neonatal (n = 1), infant (n = 37), juvenile, 1 year to 3 years (n = 30), and adult (n = 8). No geriatric animals were identified in the group that was treated in the ONPRC colony clinic and successfully released from clinical care. Table 1 summarizes the number of animals, presenting either for clinical treatment or necropsy, that cultured positive for *Y. pseudotuberculosis*, *Y. enterocolitica*, or *Y. kristensenii*, according to age, sex, and season at time of microbial culture. Table 2 notes the clinical signs identified at initial presentation in order of frequency of occurrence for animals infected with *Y. pseudotuberculosis*, *Y. enterocolitica*, or *Y. kristensenii* and successfully released from clinical care. The most common findings included diarrhea, dehydration (determined by decrease in skin turgor, dry/tacky mucous membranes and/or sunken eyes), and azotemia. 

Necropsy Findings:

*Yersinia* spp. were isolated at time of necropsy in 25 animals between the years 2000 and 2024. *Y. pseudotuberculosis* was isolated from 18 (72.0%) of these cases, while *Y. enterocolitica* was isolated from 7 (28.0%). Of the cases submitted for necropsy, 17 were females (68.0%), and 8 were males (32.0%), ranging in age from 1 day to 19 years. The age groupings included neonatal (n = 1), infant (n = 12), juvenile (n = 9), adult (n = 2), and geriatric (n = 1). Table 1 contains a summary of animals in each group (culture positive for *Y. pseudotuberculosis* vs. *Y. enterocolitica*) according to age, sex, and seasonality at time of microbial culture and necropsy.

Half of the necropsied animals culturing positive for *Y. pseudotuberculosis* were submitted following spontaneous death. An additional 5% succumbed to the disease within 48 h of clinical presentation, despite supportive and therapeutic care. Co-organisms, including *Campylobacter coli*, *Campylobacter jejuni*, *Trichuris* sp., flagellates, and *Balantidium coli* were identified in 50% (n = 9) of these cases. 14% of the cases that cultured positive for *Y. enterocolitica* at time of necropsy had been found deceased. All other animals infected with *Y. enterocolitica* presented to the clinic with diarrhea prior to necropsy. Two of the clinical cases (33%) died spontaneously following initial clinical care. Co-organisms were identified following necropsy in 85% of *Y. enterocolitica* cases, including *Campylobacter coli* (71%), *Campylobacter jejuni*, and *Shigella flexneri*.

**Figure 1:**
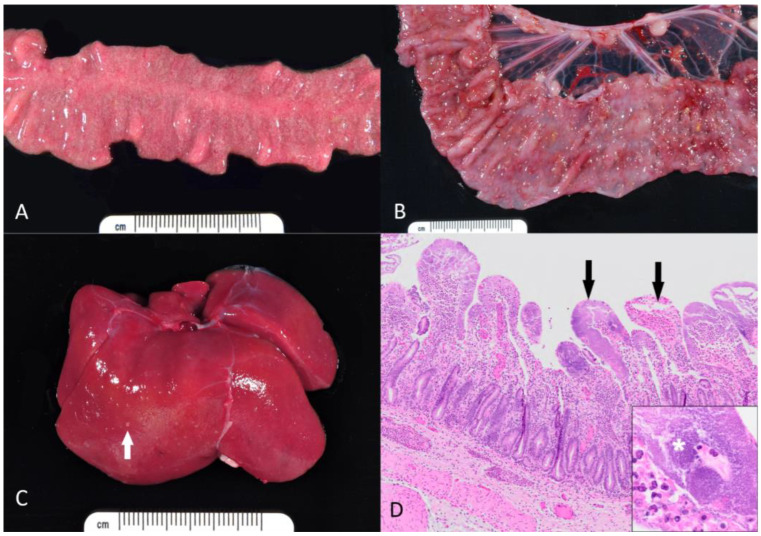
Gross and microscopic appearance of yersiniosis in rhesus macaques. (**A**) Small intestine. The mucosa is diffusely edematous and contains myriads of pale necrotic and hemorrhagic foci. (**B**) Colon. Within the mucosa, there are numerous necroulcerative foci covered with tan fibrin and rimmed by hemorrhage. (**C**) Liver. Numerous pale microabscesses (white arrow) are scattered throughout the hepatic parenchyma. (**D**) Small intestine. The lamina propria of most villi is effaced by large aggregates of coccobacilli, neutrophils, and necrotic debris (black arrows). Inset: Dense colonies of *Yersinia* bacilli (asterisk). Hematoxylin and eosin.

Systems and organs affected:

Of those cases presented to necropsy, *Y. pseudotuberculosis* was isolated on microbial culture in the small intestine (n = 7, 38.9%), colon (n = 8, 44.4%), liver (n = 4, 22.2%), blood (n = 2, 11.1%), feces (n = 2, 11.1%), mesenteric lymph nodes (n = 1, 5.6%), and lung (n = 1, 5.6%). *Y. enterocolitica* was isolated on microbial culture in the small intestine (n = 2, 28.6%), colon (n = 4, 57.1%), and feces (n = 2, 28.6%). Grossly, lesions varied from extensive involvement of multiple organs to minimal lesions in the small or large intestine. In the gastrointestinal tract, mucosal hyperemia, edema and multifocal erosions and ulcerations were observed (Figure 1A,B). Hepatic involvement was usually manifested as numerous pale foci (Figure 1C). 

Histologically, coccobacilli (*Y. pseudotuberculosis* based on culture results) were noted in the small intestine (n = 12, 66.7%), colon (n = 10, 55.6%), liver (n = 3, 16.7%), stomach (n = 2, 11.1%), spleen (n = 1, 5.6%), and mesenteric lymph nodes (n = 1, 5.6%). Histologically, coccobacilli (*Y. enterocolitica* based on culture results) were noted in the colon (n = 6, 85.7%) and small intestine (n = 4, 57.1%). Common histopathological findings included large foci of mucosal necrosis and local infiltration of inflammatory cells, especially neutrophils, forming abscesses in the lamina propria, often near Peyer’s patches. In the intestines, colonies of Gram-negative coccobacilli were characteristic (Figure 1C). Additional findings included multifocal, acute and necrotizing hepatitis, splenitis, and lymphadenitis Figure 1D. A summary of all histologic findings, in order by frequency of occurrence, can be found in Table A1 of Appendix A.

Statistical Analysis: 

Mortality occurred in 25 of 99 cases with confirmed yersiniosis (25.3%). The mortality rate was 23.7% (18/76) for those with *Y. pseudotuberculosis* infections and 30.4% (7/23) for those with *Y. enterocolitica* infections. Patients with *Y. enterocolitica* had a 28% higher risk of death compared with those infected by *Y. pseudotuberculosis* (RR 1.28, 95% CI 0.60–2.73). By two-sided Fisher’s exact test, the difference in mortality between the two species was not statistically significant (*p* = 0.589). The corresponding odds ratio was 1.41 (95% CI 0.53–3.76). 

Among the cases presented for necropsy, spontaneous death occurred in 10 animals. The spontaneous death rate was higher in animals with *Y. pseudotuberculosis* than *Y. enterocolitica* (50.0% [9/18] vs. 14.3% [1/7]). Rhesus macaques with *Y. enterocolitica* had a 78% lower risk of spontaneous death compared with those infected with *Y. pseudotuberculosis* (RR 0.22, 95% CI 0.03–1.49). By two-sided Fisher’s exact test, this difference was not statistically significant (*p* = 0.204). The corresponding odds ratio was 0.17 (95% CI 0.02–1.54). 

Co-infection with other pathogens was documented in 15 of the 25 cases presented for necropsy (60.0%). The frequency of co-infection was higher in animals with *Y. enterocolitica* than *Y. pseudotuberculosis* (85.7% [6/7] vs. 50.0% [9/18]). Cases with *Y. enterocolitica* had a 71% higher risk of co-infection compared with those infected with *Y. pseudotuberculosis* (RR 1.71, 95% CI 0.99–2.98). By two-sided Fisher’s exact test, this difference was not statistically significant (*p* = 0.179). The corresponding odds ratio was 6.00 (95% CI 0.60–60.38). 

## 4. Discussion

We identified 101 cases of culture-positive yersiniosis in rhesus macaques at the ONPRC between 2000 and 2024. Of these 101 animals, *Yersinia pseudotuberculosis* accounted for the majority (n = 76, 75.2%), while *Y. enterocolitica* was less common (n = 23, 22.8%). Based on previous data identifying a higher prevalence of *Y. pseudotuberculosis* versus *Y. enterocolitica* in our rhesus macaque colony, we suspected that morbidity and mortality would be higher in those animals infected with *Y. pseudotuberculosis* versus *Y. enterocolitica*. However, despite its higher prevalence, *Y. pseudotuberculosis* infections resulted in a slightly lower mortality rate than *Y. enterocolitica* infections (23.7% [18/76] vs. 30.4% [7/23]; *p* = 0.589). The overall mortality among the 99 culture-positive cases was 25.3%. 

*Y. kristensenii* is commonly categorized as a *Y. enterocolitica*-like species. While it has been recently studied due to its role in foodborne illness and zoonotic potential, it is widely considered non-pathogenic [28]. Because the two rhesus macaques in our dataset that cultured positive for *Y. kristensenii* presented with limited clinical signs and survived to release without complication, its implications in the ONPRC colony were not evaluated for this study. However, some new research has been performed to better characterize the genotypic and phenotypic virulence potential of *Y. enterocolitica*-like species (*Y. kristensenii*, *Y. frederiksenii*, and *Y. intermedia*). While typically regarded as commensal or opportunistic species, the possibility of strain-specific pathogenicity could not be excluded. Some analyses suggest that *Y. kristensenii* possesses several virulence-associated genes, though their functional expression and clinical relevance remain uncertain [29,30]. Continued monitoring and characterization of isolates will be important to determine whether *Y. kristensenii* plays a more meaningful role in the pathogenicity of yersiniosis in NHPs than previously appreciated. 

In our population of rhesus macaques at the ONPRC, the clinical presentations of animals infected with *Y. pseudotuberculosis* or *Y. enterocolitica* included signs of chronic enteric disease (dehydration, diarrhea, weight loss). Azotemia was noted clinically, and renal tubular necrosis was identified on microscopic examination in most cases. These findings were attributed to diarrhea-related dehydration and renal hypoperfusion. *Y. pseudotuberculosis* was cultured outside of the GI tract, including the liver, blood, and mesenteric lymph nodes, indicating a higher likelihood of systemic lesions compared to *Y. enterocolitica*, which was only cultured from the small intestine and colon. Histologically, coccobacilli (*Y. pseudotuberculosis* based on culture) were identified multiple tissues, including the small intestine, colon, liver, stomach, spleen, and mesenteric lymph nodes, in contrast to *Y. enterocolitica*, which could only be identified histologically in the small intestine and colon. Spontaneous death (animals found deceased without prior clinical presentation) was uncommon overall but occurred more often in macaques infected with *Y. pseudotuberculosis* than *Y. enterocolitica* (50.0% vs. 14.3%). Although *Y. enterocolitica* infections showed a lower relative risk of spontaneous death (RR 0.22; 95% CI 0.03–1.49) and a lower odds ratio (OR 0.17; 95% CI 0.02–1.54), the finding was not statistically significant (*p* = 0.204). Overall, the data suggest a trend toward higher spontaneous mortality with *Y. pseudotuberculosis*, but the small sample size limits firm conclusions. 

Our findings highlight important differences in disease patterns between the two primary *Yersinia* spp. *Y. pseudotuberculosis* was strongly associated with systemic infection: isolates were recovered from extra-intestinal sites including the liver, blood, and mesenteric lymph nodes, and histologic examination confirmed widespread dissemination of coccobacilli. These infections were frequently identified in animals found dead or succumbing rapidly after clinical presentation, supporting its role as a potential cause of spontaneous (sudden) death in rhesus macaques. In contrast, *Y. enterocolitica* was strictly isolated from the gastrointestinal tract and produced a more localized enteric disease pattern, often characterized by diarrhea, dehydration, and weight loss. Although *Y. enterocolitica* cases had a slightly higher mortality rate, this trend did not reach statistical significance. This could be due to the limited number of affected animals, but may also be multifactorial.

Coinfections, especially *Campylobacter* spp., were frequent, particularly in cases of *Y. enterocolitica*. These were identified in 85% of *Y. enterocolitica*-positive necropsy cases, compared with 50% of *Y. pseudotuberculosis* cases. However, the lack of statistical significance (*p* = 0.179) indicates that these findings should be interpreted cautiously, as the small number of *Y. enterocolitica* cases may inaccurately amplify perceived differences and prevent definitive conclusions. This disparity may suggest that *Y. enterocolitica* can act as an opportunistic pathogen or that gastrointestinal coinfections exacerbate disease severity, but further investigation with a larger study group is warranted [31,32,33,34]. It is also important to note that several of the enteric pathogens identified as co-infections are more commonly detected within the ONPRC colony than *Yersinia* spp. For example, over the study period, *C. coli* was isolated from 8232 rhesus macaque samples, and *C. jejuni* was isolated from 888 rhesus macaque samples. These background detection rates suggest that the presence of co-infection may reflect common microbial carriage rather than a direct causal relationship with disease severity.

To summarize, several conclusions can be drawn from the data gathered, each addressing different aspects of infection severity and prevalence associated with *Y. pseudotuberculosis* and *Y. enterocolitica* in rhesus macaques. When considering infection severity, we recognized that, while *Y. pseudotuberculosis* was isolated more commonly overall, a non-significant trend towards lower mortality was observed. Although not statistically significant, *Y. pseudotuberculosis* infections were associated with a higher proportion of animals dying spontaneously before exhibiting clinical signs, whereas *Y. enterocolitica* cases showed a trend toward higher overall mortality but tended to develop signs of illness that warranted clinical intervention. Multiorgan involvement may explain the trend towards higher rates of spontaneous death associated with *Y. pseudotuberculosis* infection [35]. Coinfections, particularly with *Campylobacter* spp., were more frequent in animals culturing positive for *Y. enterocolitica* than with *Y. pseudotuberculosis*. The trend towards higher mortality in *Y. enterocolitica* cases could be partly attributed to the combined pathogenic impact of mixed infections. This may imply that *Y. enterocolitica* is opportunistic or requires cofactors to cause severe disease [33]. The clinical and diagnostic implications of these findings suggest that *Y. pseudotuberculosis* may need to be considered in cases of unexplained sudden death or multisystemic signs. Additionally, animals with severe clinical signs associated with *Y. enterocolitica* infections might warrant investigation for coinfection, particularly when gastrointestinal signs are present. 

The influence of age and immune maturity should also be considered. Infants and juveniles represented the largest portion of affected animals in this study. Young rhesus macaques may be particularly vulnerable due to immature immune defenses, social contact within age-matched juvenile groups, and increased exposure due to environmental exploration, all of which elevate the likelihood of exposure and clinical disease. While it is uncertain, due to the retrospective nature of this study, transmission from subclinical dams to their infants may have played a role in the increased rate of infection in the neonatal and infant age groups. Adults and geriatric animals were comparatively underrepresented, suggesting some degree of acquired immunity or reduced exposure risk [21,31,36].

The environmental and seasonal trends identified in this study are consistent with the psychrotrophic nature of *Yersinia* spp., which are capable of persisting in soil, water, and food sources at low temperatures [31,32]. Outdoor housing, where animals are exposed to fluctuating weather and potential wildlife reservoirs, appears to represent a key risk factor for transmission [37,38]. Specifically, the cold temperatures and heavy rainfall, common during Oregon winters, can lead to longer exposure to wet ground cover [39,40]. Seasonal peaks may also coincide with changes in NHP behavior, such as increased clustering for warmth, which can facilitate pathogen spread within social groups [36,41]. Additionally, rodent and avian populations, recognized reservoirs of *Yersinia*, often increase their interactions with outdoor enclosures during cooler months, further elevating exposure risk [21,31]. These observations highlight the need for continued disease surveillance that considers not only colony management practices but also local wildlife and environmental monitoring. 

While our findings suggest that *Y. pseudotuberculosis* may be more prevalent and commonly associated with systemic disease in rhesus macaques compared to *Y. enterocolitica*, several limitations must be considered when interpreting these results. Isolating and culturing *Yersinia* spp. from clinical samples can be challenging. The phenotypic API20E method for species identification is not the most reliable system for differentiating between *Yersinia* spp., especially non-enterocolitica *Yersinia* [42,43] [Varettas and Mancini]. False-negative culture results may occur, especially in cases with a low bacterial load or bacterial loads that fluctuate due to intermittent shedding, when *Yersinia* is outcompeted by other microbial flora, or when samples are obtained after antibiotic administration [7,44]. Previous studies have demonstrated variable sensitivity of *Yersinia* cultures depending on methodology and timing of specimen collection [45,46].

Additionally, histopathologic evidence suggestive of *Yersinia* infection was identified in several cases in the absence of confirmatory microbial culture. These cases often included gross lesions consistent with *Yersinia* infection (e.g., mesenteric lymphadenitis, enterocolitis) and coccobacilli observed microscopically; however, definitive microbial identification was not obtained. The identification of histopathologic evidence suggestive of *Yersinia* infection in the absence of confirmatory microbial culture has several important consequences. First, it highlights the diagnostic limitations of relying solely on culture-based methods. *Yersinia* spp. can be difficult to isolate due to intermittent shedding, competition with commensal flora, or prior antimicrobial therapy, as previously mentioned [38,47]. As a result, some true cases of yersiniosis may have been excluded from our dataset, potentially skewing calculations. From a colony health management perspective, under-recognition of *Yersinia* infections could delay or complicate outbreak responses. Animals with suggestive clinical signs or lesions but no culture confirmation might not have been classified as cases, potentially obscuring patterns of transmission within the colony. This potential underestimation of disease prevalence could lead to an underappreciation of the pathogen’s role in morbidity, mortality, or reproductive loss. 

Finally, the retrospective nature of this study introduces several important limitations that influence interpretation of the findings [48,49]. Because diagnostic approaches evolved over the 24-year study period, the accuracy in identifying *Yersinia* infections was likely inconsistent. For example, earlier records may have relied more heavily on gross pathology or culture under suboptimal conditions, while later years benefited from improved microbiological techniques and record-keeping. This variation creates the possibility of temporal bias, in which the apparent prevalence of yersiniosis reflects changes in diagnostic capability rather than true shifts in disease burden. Inconsistent sample collection must also be considered [48]. Some animals may have undergone full diagnostic workups, while others may have been sampled more selectively depending on clinical presentation or logistical constraints. This non-uniformity can lead to underdiagnosis in mildly affected or subclinical animals and overrepresentation of severe cases. In the future, the epidemiology and clinical significance of yersiniosis could be better characterized by utilizing data that have been gathered following standardized diagnostic criteria and uniform sampling protocols within the NHP colony [48,49]. 

## 5. Conclusions

This retrospective study offers insights into the epidemiology and pathogenicity of *Yersinia* infections in a captive rhesus macaque colony. *Y. pseudotuberculosis* was more frequently isolated and more often linked to systemic disease and spontaneous death, while *Y. enterocolitica* exhibited a slightly higher, although statistically non-significant, overall mortality rate and was more commonly associated with concurrent infections, particularly *Campylobacter* spp. These findings underscore the need for continued diagnostic surveillance and prospective research to better understand the role of *Y. pseudotuberculosis* in sudden, multisystemic disease and the contribution of coinfections as they pertain to the severity of *Y. enterocolitica* infections. 

From a laboratory animal medicine perspective, these results emphasize the importance of ongoing diagnostic surveillance, especially in outdoor-housed primates during cooler seasons when *Yersinia* infections are more prevalent. The identification of culture-negative but histologically consistent cases also highlights the limitations of culture-based diagnostics. Molecular assays such as multiplex PCR have shown promise in improving detection rates and differentiating pathogenic strains and could be considered in the future depending on availability [38]. Beyond colony health, the findings have broader relevance to zoonotic risk assessment and comparative medicine. Many of the clinical signs reported in NHPs mirror patterns reported in human yersiniosis, supporting the rhesus macaque as a valuable model for understanding disease dynamics across species [7,35,50]. Finally, this study underscores the need for prospective research incorporating standardized diagnostic protocols, pathogen detection, and systematic evaluation of potential risk factors. Such approaches will be critical for refining disease control strategies, protecting colony health and research integrity, and clarifying the zoonotic significance of *Yersinia* spp. in NHP populations. 

## Figures and Tables

**Table 1:** Demographics of Rhesus Macaques Culturing Positive for *Yersinia* spp. (Clinical vs. Necropsy Cases).

Demographics		*Y. pseudotuberculosis*		*Y. enterocolitica*		*Y. kristensenii*		Total
		Clinical	Necropsy	Clinical	Necropsy	Clinical	Necropsy	
Sex	Female	34	13	7	4	2	0	60
	Male	24	5	9	3	0	0	41
Age Category	Neonate	0	1	1	0	0	0	2
	Infant	33	11	4	1	0	0	49
	Juvenile	21	4	8	5	1	0	39
	Adult	4	1	3	1	1	0	10
	Geriatric	0	1	0	0	0	0	1
Season at Time of Microbial Culture	Winter (Dec.–Feb.)	35	13	4	6	0	0	58
	Spring (March–May)	20	2	9	0	2	0	33
	Summer (June–Aug.)	0	0	1	0	0	0	1
	Fall (Sept.–Nov.)	3	3	2	1	0	0	9

**Table 2:** Common Clinical Presentations for Rhesus Macaques Treated and Released from the Colony Hospital.

Clinical Presentation	Organism			Total
	*Y. pseudotuberculosis*	*Y. enterocolitica*	*Y. kristensenii*	
Diarrhea	53	10	1	67
Dehydration	53	10	1	67
Azotemia	37	7	0	44
Poor BCS/weight loss	10	4	0	14
Colonic distention	9	1	1	11
QAR mentation	4	1	0	5
Trauma	1	3	0	4
No Clinical Signs	1	2	1	4
Hypothermia	0	3	0	3
Hunched/painful	2	0	0	2
Lymphadenomegaly	0	1	1	2
Muscle wasting	0	1	0	1
Lethargy	0	1	0	1
CBC abnormality	1	0	0	1

## Data Availability

The original contributions presented in this study are included in the article. Further inquiries can be directed to the corresponding author.

## Abbreviations

The following abbreviations are used in this manuscript:

ONPRC	Oregon National Primate Research Center
NHP	Nonhuman Primate

## Appendix A

**Table A1:** Common Histologic Findings in Rhesus Macaques Submitted for Necropsy Following Infection with *Y. pseudotuberculosis* or *Y. enterocolitica*.

Organ/System	Histologic Findings	Organism		Total
		*Y. pseudotuberculosis*	*Y. enterocolitica*	
Small Intestine	Inflammation	10	4	14
	Neutrophilic infiltration	7	2	9
	GALT depletion	3	1	4
	GALT hyperplasia	2	0	2
	Crypt hyperplasia	2	0	2
	Amyloid	2	6	8
	Crypt abscess	0	1	1
Colon	Inflammation	13	7	20
	Neutrophilic infiltration	8	2	10
	Amyloid	1	0	1
	Crypt abscess	0	2	2
Cecum	Inflammation	10	7	17
Kidney	Hyaline casts	2	2	4
	Tubular necrosis	12	4	16
	Inflammatory infiltrate	1	1	2
	Amyloid	1	0	1
Mesenteric LN	Inflammation	4	0	4
	Lymph follicle depletion	4	1	5
	Lymphoid hyperplasia	3	1	4
	Fibrin deposition	1	0	1
	Amyloid	1	1	2
Stomach	Inflammation	5	1	6
	Neutrophilic infiltration	2	0	2
	Amyloid	1	4	5
Thymus	Depletion	10	3	12
	Amyloid	1	0	1
Spleen	Inflammation	2	0	2
	Lymph follicle depletion	4	1	5
	Amyloid	1	4	5
	Lymphoid follicle hyperplasia	1	0	1
Liver	Inflammation	6	0	6
	Micro abscess	1	0	1
	Inflammatory infiltrate, portal tract	1	0	1
	Amyloid	2	1	3
Peripheral LN	Lymph follicle depletion	4	2	6
	Hyperplasia	1	1	2
	Inflammation	1	0	1
	Amyloid	1	1	2
Joint	Synovial inflammation	3	1	4
Lung	Neutrophilic infiltration	2	0	2
	Edema, congestion	2	0	2
Adrenal gland	Congestion, cortex	2	0	2
	Amyloid	1	0	1
	Hemorrhage	1	0	1
Cerebrum	Leptomeningeal edema	3	0	3
Heart	Amyloid	1	0	1
	Inflammation	1	0	1
Gallbladder & Bile Ducts	Inflammation	1	0	1

## Appendix B

**Table A2:** *Yersinia* spp. Culture Results by Year (2000–2024).

		Necropsy *Y. pseudotuberculosis*				Necropsy *Y. enterocolitica*				Clinical *Y. pseudotuberculosis*				Clinical *Y. enterocolitica*				Clinical *Y. kristensenii*
Year	TOTAL	Winter	Spring	Summer	Fall	Winter	Spring	Summer	Fall	Winter	Spring	Summer	Fall	Winter	Spring	Summer	Fall	Spring
2000	1	1																
2001	3							1		1						1		
2002	4		2												1			1
2007	5					1									4			
2009	4		1											3				
2011	3									1							1	1
2012	3	2			1													
2013	8	2				2	1			1					2			
2015	4	2											1				1	
2016	2					1			1									
2017	3	1	1							1								
2019	3	1			1					1								
2020	6		1							4			1					
2021	16				1					9	5		1					
2022	6									5				1				
2023	24									7	15				2			
2024	6	1								5								
TOTALS	101	10	5	0	3	4	1	1	1	35	20	0	3	4	9	1	2	2
